# Light Capture, Skeletal Morphology, and the Biomass of Corals’ Boring Endoliths

**DOI:** 10.1128/mSphere.00060-21

**Published:** 2021-02-24

**Authors:** A. J. Fordyce, T. D. Ainsworth, W. Leggat

**Affiliations:** a School of Environmental and Life Sciences, University of Newcastle, Ourimbah, NSW, Australia; b School of Biological, Earth and Environmental Sciences, University of New South Wales, Sydney NSW, Australia; University of Wisconsin—Madison

**Keywords:** chlorophyll, coral reef, coral skeleton, endolith, microbial biomass, microhabitat, *Ostreobium*

## Abstract

There is a growing interest in the endolithic microbial biofilms inhabiting skeletons of living corals because of their contribution to coral reef bioerosion and the reputed benefits they provide to live coral hosts. Here, we sought to identify possible correlations between coral interspecific patterns in skeletal morphology and variability in the biomass of, and chlorophyll concentrations within, the endolithic biofilm. We measured five morphological characteristics of five coral species and the biomasses/chlorophyll concentrations of their endolithic microbiome, and we compare interspecific patterns in these variables. We propose that the specific density of a coral’s skeleton and its capacity for capturing and scattering incident light are the main correlates of endolithic microbial biomass. Our data suggest that the correlation between light capture and endolithic biomass is likely influenced by how the green microalgae (obligatory microborers) respond to skeletal variability. These results demonstrate that coral species differ significantly in their endolithic microbial biomass and that their skeletal structure could be used to predict these interspecific differences. Further exploring how and why the endolithic microbiome varies between coral species is vital in defining the role of these microbes on coral reefs, both now and in the future.

**IMPORTANCE** Microbial communities living inside the skeletons of living corals play a variety of important roles within the coral meta-organism, both symbiotic and parasitic. Properly contextualizing the contribution of these enigmatic microbes to the life history of coral reefs requires knowledge of how these endolithic biofilms vary between coral species. To this effect, we measured differences in the morphology of five coral species and correlate these with variability in the biomass of the skeletal biofilms. We found that the density of the skeleton and its capacity to trap incoming light, as opposed to scattering it back into the surrounding water, both significantly correlated with skeletal microbial biomass. These patterns are likely driven by how dominant green microalgae in the endolithic niche, such as *Ostreobium* spp., are responding to the skeletal morphology. This study highlights that the structure of a coral’s skeleton could be used to predict the biomass of its resident endolithic biofilm.

## INTRODUCTION

Marine endolithic (i.e., living within rock) microbial biofilms are cosmopolitan in their distribution and inhabit a wide range of substrates, including the shells and skeletons of calcifying organisms such as hard corals, bivalves, and foraminifera (1–4). The microbes in these communities can be subdivided based on their niche endolithic lifestyle (1): euendoliths actively and chemically bore into rock, cryptoendoliths grow within structural cavities within porous rocks, and chasmoendoliths inhabit fissures and cracks. Although they are not active borers, cryptoendoliths and chasmoendoliths nonetheless degrade substrates through the alteration of pore water chemistry (i.e., biocorrosion) (5–7). When these organisms colonize the shell or skeleton of a marine calcifier, they can be parasitic and reduce the survival and growth of the host by degrading the its protective shell (3, 8) or creating lesions (9, 10). But they can also be beneficial, for example, by offsetting high temperature or light stress to the host (11–13) or by providing nutrients during times of environmental stress (14–16). This dual capacity for symbiosis and parasitism (12) underlies the growing interest in the potential role of endolithic biofilms in the future sustainability of marine calcifiers under climate change (3, 17–19).

The influence of endolithic biofilms on host responses to environmental change is of keen interest for research focusing on hard corals (Scleractinia, Cnidaria) which act as ecosystem engineers for tropical coral reefs (17, 18, 20, 21). The endolithic microbiome of hard corals is diverse and composed of both eukaryotic (microalga and fungi) and prokaryotic (cyanobacteria, bacteria, and archaea) microbes, as well as marine viruses (18, 22). Evidence to date indicates that the biomass of these microbial assemblages is predominantly composed of euendolithic siphonous unicellular algae in the genus *Ostreobium* (17, 21). Microfungi (4, 23) and cyanobacteria are also important bioeroders and often the pioneer colonizers of newly denuded substrates (24). Other important functional groups within the coral endolithic microbiome include diazotrophic and other bacterial taxa (25) which participate in nitrogen and phosphorous regeneration of the skeletal pore water (26, 27). Given their capacity to affect the health of corals as keystone species, coral endolithic microbes can affect whole ecosystem function. Endolithic biofilms in dead coral substrates have previously been identified as significantly contributing to whole-reef primary productivity (28). This is in addition to being major contributors to reef bioerosion, total carbonate budgets and bathymetric structural complexity (29–31). Endolithic microfungi have also been observed parasitizing their cnidarian hosts (32), which could reduce coral fitness. As such, the coral endolithic microbiome can be parasitic and symbiotic to its host, providing the coral with nutrients but simultaneously damaging their supportive skeleton, and through these behaviors they can affect whole ecosystem characteristics.

Variation in the macro- and microstructure of the coral skeleton is often highlighted as a potentially important factor affecting endolithic microbial biomass and the contribution of microbial endoliths to coral health and degradation (33–36). Vogel et al. (36) previously showed that the rate of microbial bioerosion varies between types of calcifier shells as well as between different mineral phases of calcium carbonate (e.g., calcite versus micrite). For hard corals, there are many possible host skeletal characteristics to which endolithic biofilms may respond, but interspecific variability in endolithic community composition and biomass is not well known. For example, massive or mounding corals, whose microbial endoliths are most commonly studied, have a large volume of substrate to colonize which could lead to greater colony-specific biomass for these corals. However, the quality of the substrate, as well as quantity, needs to be considered. Ralph et al. (37) note that endolithic microalgae within corals from reef lagoons with high irradiance show contrasting behavior to those from deeper reef slopes (38). Namely, microborers burrow downwards (i.e., positive geotropism) in high irradiance habitats while they grow “upward” toward the host tissue in deeper habitats. This suggests that the compensation depth for photosynthetically active radiation inside the coral skeleton varies across habitats. Therefore, a massive coral on a deep reef may have a large absolute volume of substrate, but only a small portion of this can support the phototrophic growth of dominant microalgae in the skeleton. In contrast, tabular corals have evolved to maximize light capture for the endosymbiotic algae within their tissues (i.e., zooxanthellae [39]); this may also increase the internal skeletal light field in the endolithic microenvironment. An expected result may be a higher endolithic biomass compared to massive corals but in high irradiance habitats, light enhancement may lead to photodamage/photoinhibition in *Ostreobium* spp. adapted to extreme shade but lacking typical photoprotective mechanisms designed to prevent damage from excess light (37, 40, 41). Therefore, tabular coral skeleton may in fact be a poor-quality habitat on reef flats but a good-quality habitat on reef slopes.

Variation in coral biomechanical and morphological features has been previously shown to correlate with the diversity of coral-associated bacterial communities (42, 43). For the endolithic component of the coral microbiome, interspecific variability in coral skeletal porosity and density are expected to affect the biomass of the resident endolithic biofilm. Crypto- and chasmoendolithic taxa may benefit from high porosity since it represents greater available space for colonization. Euendolithic taxa would, in theory, be more sensitive to interspecific variability in skeletal microdensity since it is expected to effect the energetic cost of boring into the coral skeleton. Variation in coral skeletal density arises from (i) the organic matrix intercalating the skeleton, (ii) aragonite grain size and orientation, and (iii) small amounts of nonaragonitic calcium carbonates such as calcite and micrite. Vogel et al. (36) demonstrated that bioerosion rates differ between aragonite, calcite, and micrite, while Iha et al. (41) recently presented data that suggests euendolithic *Ostreobium* feeds on coral organic matrices. As such, evidence suggests that coral microdensity is a key skeletal characteristic affecting euendolithic taxa distribution and therefore overall microbial biomass inside the skeleton. Our ability to model the impact of endolithic microbiomes on whole coral reefs, however, is limited by an incomplete understanding of the nature and drivers of interspecific variability in the coral endolithic microbiome.

**FIG 1:**
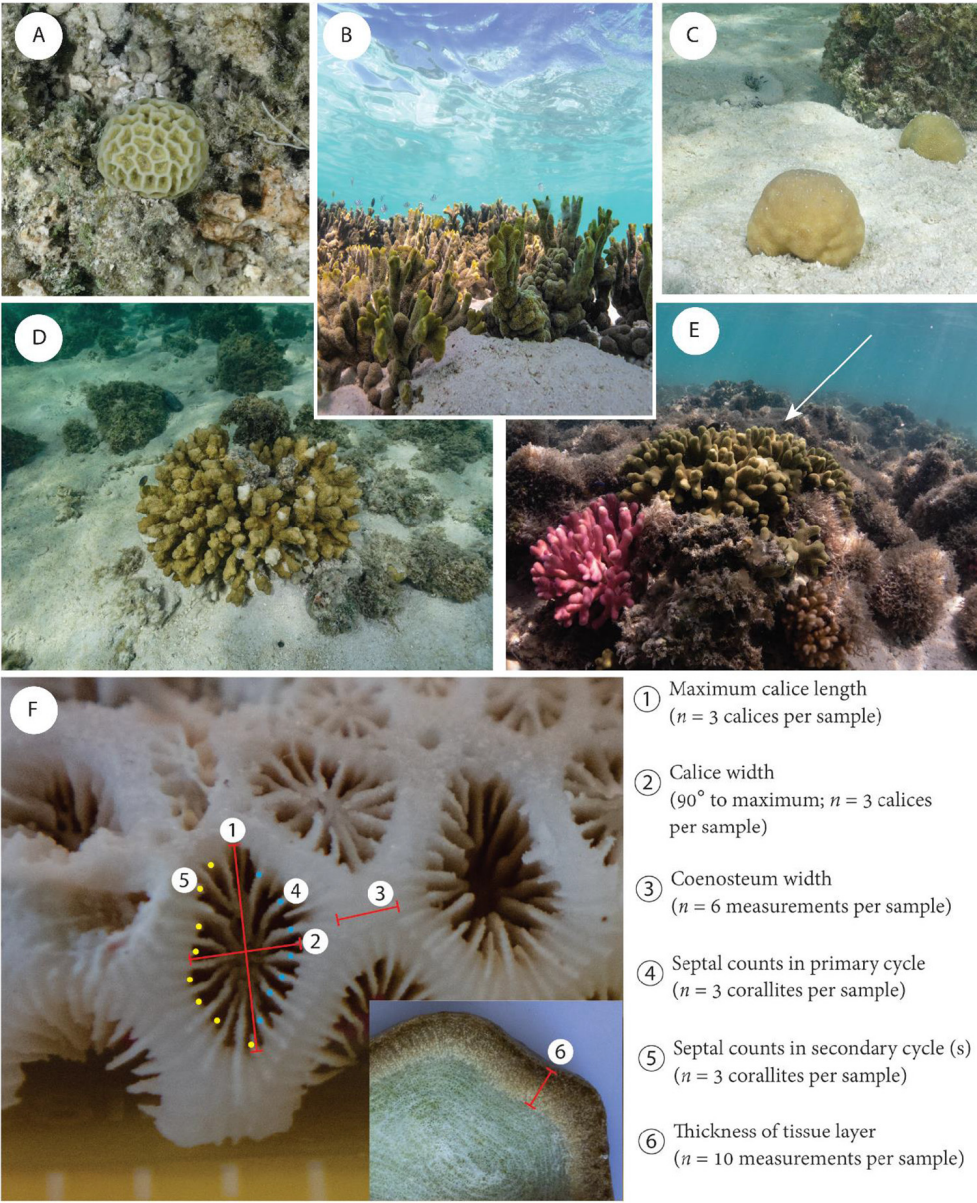
Coral species selected for this study: *Goniastrea retiformis* (A), *Montipora digitata* (B), *Porites* cf. *mayeri* (C), *Isopora palifera* (D), and *Porites cylindrica* (E; white arrow). (F) For each coral species, five morphological parameters were measured for their hypothesized influence upon endolithic microbial biomass. Measurements 1 to 3 are used to calculate the ratio of calice width to coenosteum width; measurements 4 and 5 are used to calculate corallite complexity. Tissue thickness is measured from a skeletal cross-section (inset). For details on measurements of microdensity and porosity, see Fig. S2 and Text S1. Photographs A and C to F were provided by Francesco Ricci and are used with permission.

Here, we aimed to test whether coral skeletal characteristics correlate with variability in the biomass and chlorophyll concentrations of the endolithic microbiome of five species of tropical hard coral (Fig. 1): *Goniastrea retiformis*, *Isopora palifera*, *Montipora digitata*, *Porites cylindrica*, and *Porites* cf. *mayeri*. We measured two skeletal porosity and microdensity, as well as two microstructural elements which can affect light capture/scattering on the surface of the coral colony: the size of calices relative to intercorallite space and corallite complexity (Fig. 1) (44). Based on previous studies of coral light capture (44–46), larger and more complex corallites are considered more effective “photon traps,” which increases light availability for tissue-associated zooxanthellae. Finally, we measured tissue thickness (Fig. 1) according to the hypothesis of Shashar et al. (47) that coral tissue thickness affects irradiance within the endolithic microhabitat.

10.1128/mSphere.00060-21.1 TEXT S1 Output of diagnostic tests applied to statistical models used in this study. Download Text S1, PDF file, 0.1 MB. Copyright © 2021 Fordyce et al. 2021 Fordyce et al. https://creativecommons.org/licenses/by/4.0/ This content is distributed under the terms of the Creative Commons Attribution 4.0 International license.

Simultaneously, we measured the biomass of the endolithic biofilm in each coral species (by ash-free dry weight [AFDW]) and the concentrations of chlorophylls *a*, *b*, *c*, and *d* (by spectrophotometry). Chlorophyll *a* is the primary pigment used in oxygenic photosynthesis. Chlorophyll *b* is the major accessory pigment used by green algae (Chlorophyta) such as *Ostreobium* spp. Chlorophyll *c* (here encompassing both *c*1 and *c*2) is used by a wide range of microbial phototrophs, including dinoflagellates, diatoms, and brown algae, all of which have been previously identified from the endolithic microbiome (22). Chlorophyll *d* is the primary pigment in *Acaryochloris marina* and *Acaryochloris*-like cyanobacteria (48–50), which have been identified from endolithic habitats at this location (48). We then used principal-component regression to combine the five morphological parameters and test their relationships with the biomass of and chlorophyll concentrations within the endolithic biofilm.

## RESULTS

### Interspecific comparisons. (i) Endolithic biofilm composition.

*Ostreobium* spp., identifiable by the sporangium-like swellings on their algal filaments (16), were identified in every sample of every species of scleractinian coral examined in this study (see Table S2 in the supplemental material). As expected based upon past literature (17, 21), filamentous green algae, including but not limited to *Ostreobium* spp., were the most abundant group of microbes observed in the coral endolithic biofilms. Cyanobacteria were also observed in all the studied coral species studied here, although they were considerably less common. Presence or absence data for other identified taxa are provided in Table S2.

### (ii) Endolithic microbial biomass by ash-free dry weight.

**FIG 2:**
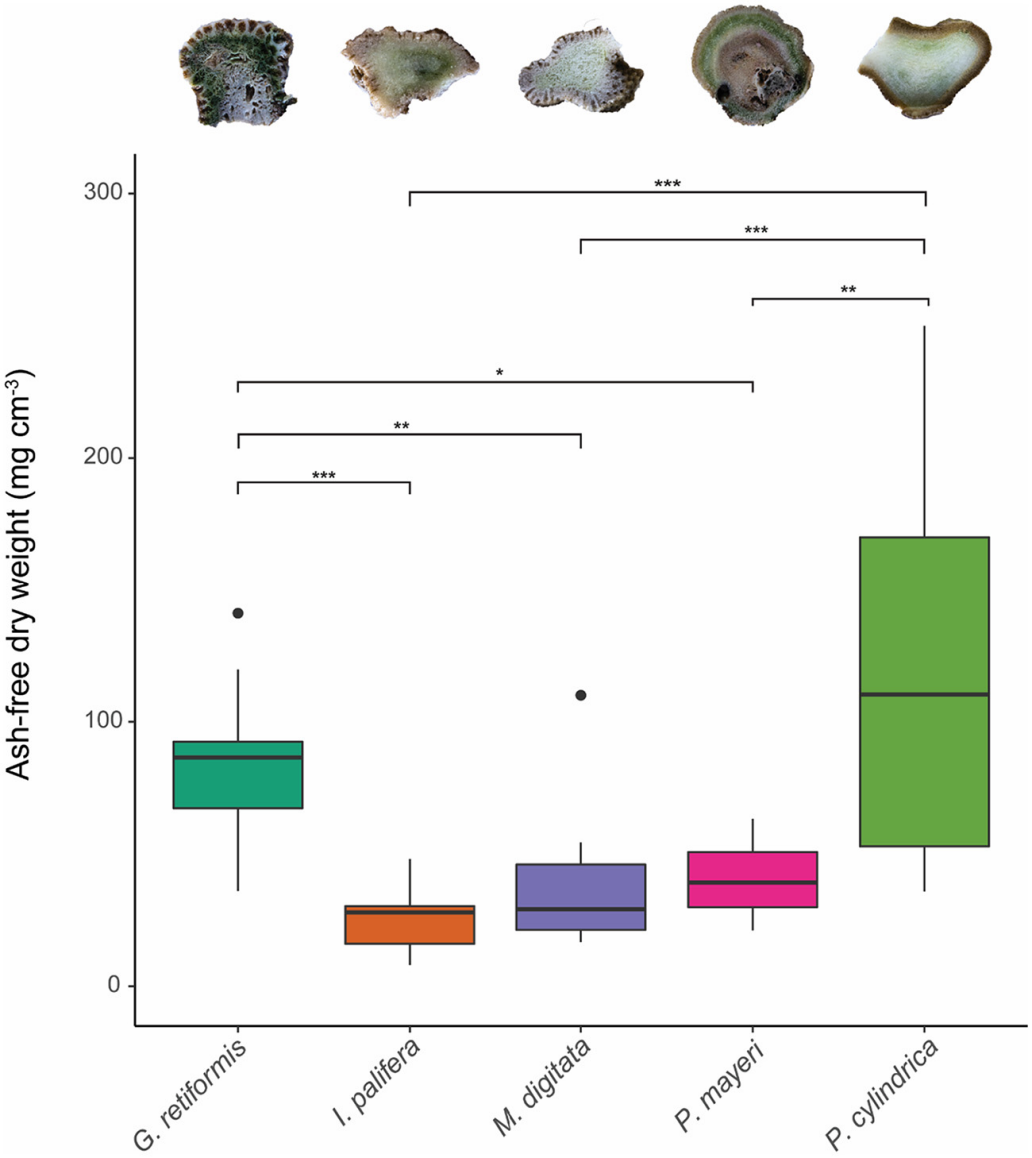
Endolithic microbial biomass measured as ash-free dry weight in mg cm^−3^ for each of the selected species (*y* axis) of tropical hard coral. Box-and-whisker plots show median and interquartile ranges (box) and the maximum and minimum (whiskers), excluding outliers (points). The insets above are cross-sectional photographs of each species corresponding to the boxplot below it; all show an endolithic green band and/or coloration. Significance: *, *P* < 0.05; **, *P* < 0.01; ***, *P* < 0.001.

Microbial biomass in the endolithic habitat was highest for *P. cylindrica* and *G. retiformis*, with median biomasses of 110.3 ± 92.923 mg cm^−3^ and 86.495 ± 22.799 mg cm^−3^, respectively (Fig. 2). These values were statistically greater than those recorded for *I. palifera* (27.899 ± 8.274 mg cm^−3^), *M. digitata* (29.102 ± 14.948 mg cm^−3^) and *P. mayeri* (39.242 ± 16.437 mg cm^−3^) (*P* < 0.05; Fig. 2; see also Table S3). *P. cylindrica* and *G. retiformis* were not significantly different to each other (*t*_4, 45_ = −0.851; *P* = 0.913; Fig. 2; see also Table S3). Similarly, none of the observed differences between *I. palifera*, *M. digitata*, and *P. mayeri* were significantly different (*P* > 0.05; Fig. 2; see also Table S3). Four outliers were identified while conducting the one-way analysis of variance (ANOVA), but excluding these did not affect model outcomes and so they were retained.

### (iii) Chlorophyll *a* concentrations.

**FIG 3:**
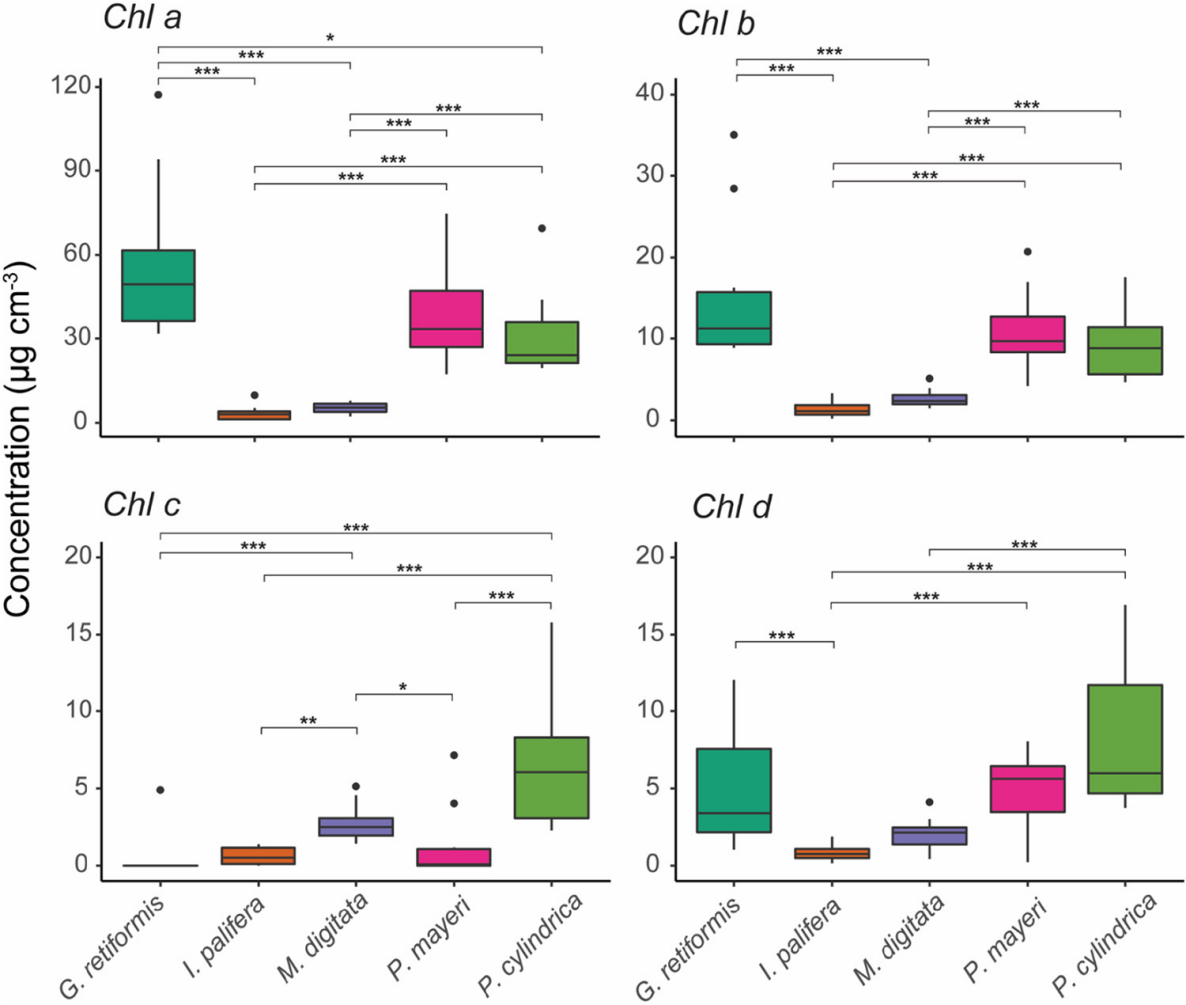
Endolithic concentrations of chlorophylls *a* to *d* (μg cm^−3^) measured using spectrophotometry, including the results of interspecific comparisons for each pigment. Box-and-whisker plots show medians and interquartile ranges (boxes) and the maximum/minimum (whiskers), excluding outliers (points). Significance: *, *P* < 0.05; **, *P* < 0.01; ***, *P* < 0.001.

*G. retiformis* skeletons had the highest median concentration of chlorophyll *a* (49.534 ± 22.968 μg cm^−3^), followed by *P. mayeri* (33.372 ± 19.757 μg cm^−3^; Fig. 3, *Chl a*). These two species were not significantly different from each other (*t*_4, 45_ = 1.994; *P* = 0.258; Fig. 3, *Chl a*; see also Table S4). *P. cylindrica* had the third highest concentration in its skeleton (24.107 ± 6.625 μg cm^−3^), followed by *M. digitata* (5.458 ± 2.261 μg cm^−3^) and finally *I. palifera* (3.059 ± 2.460 μg cm^−3^). The chlorophyll *a* concentration in the skeleton of *G. retiformis* was significantly higher than all species (*P* < 0.05) except *P. mayeri* (Fig. 3, *Chl a*; see also Table S4). Both *Porites* species had significantly greater concentrations of chlorophyll *a* than *I. palifera* and *M. digitata* (*P* < 0.05), but the poritids were not significantly different to each other (*t*_4, 45_ = 0.935; *P* = 0.882). Similarly, *I. palifera* and *M. digitata* were not significantly different (*t*_4, 45_ = −2.31; *P* = 0.161; Fig. 3, *Chl a*; see also Table S4).

The one-way ANOVA used to compare interspecific differences in chlorophyll *a* concentration had two outliers: one *I. palifera* sample (9.862 μg cm^−3^, Cook’s distance = 0.138) and one *P. cylindrica* sample (69 μg cm^−3^, Cook’s distance = 0.093). Removing these outliers altered the results of the interspecific comparisons, making the difference between *I. palifera* and *M. digitata* significant where it previously was not (*t* = −3.145, *P* = 0.024 versus *t* = −2.310, *P* = 0.161). We found no obvious biological or technical reason to exclude these data points; therefore, they were retained, and the results from the full model are presented.

### (iv) Chlorophyll *b* concentrations.

The interspecific patterns in chlorophyll *b* concentrations in the endolithic microbiome mirrored those for chlorophyll *a* (Fig. 3, *Chl b*). *G. retiformis* (11.236 ± 3.457 μg cm^−3^) had the highest concentration of chlorophyll *b* in its skeleton, followed by *P. mayeri* (9.683 ± 4.353 μg cm^−3^), *P. cylindrica* (8.833 ± 4.502 μg cm^−3^), *M. digitata* (2.379 ± 0.916 μg cm^−3^), and *I. palifera* (1.112 ± 0.921 μg cm^−3^). In regard to the statistical comparisons, the outcomes were also the same as for chlorophyll *a* (Fig. 3, *Chl b*; see also Table S4), with the exception that the chlorophyll *b* concentration in *G. retiformis* was not significantly greater than that measured in *P. cylindrica* (8.833 ± 4.502 μg cm^−3^) (*t* = 2.148, *P* = 0.2184). A single *G. retiformis* sample was the only outlier with a high pigment concentration (35.001 μg cm^−3^, Cook’s distance = 0.115).

### (v) Chlorophyll *c* concentrations.

Nine of the ten samples analyzed for *G. retiformis* had no chlorophyll *c*; the only sample in which it was detected had a concentration of 4.899 μg cm^−3^. Similarly, chlorophyll *c* was not detected in half of the *P. mayeri* samples; the other half had a median concentration of 0.081 ± 0.120 μg cm^−3^. *I. palifera* similarly had very low concentrations of chlorophyll *c*, with a median concentration of 0.515 ± 0.704 μg cm^−3^. In contrast, *M. digitata* and *P. cylindrica* had median chlorophyll *c* concentrations of 2.491 ± 0.905 μg cm^−3^ and 6.047 ± 4.213 μg cm^−3^, respectively. *P. cylindrica* and *M. digitata* both had significantly higher concentrations of chlorophyll *c* in its skeleton than other three coral species, but were not significantly different to each other (Fig. 3, *Chl c*; see also Table S4). There were three outliers in the statistical model: the aforementioned *G. retiformis* sample and two *P. mayeri* samples which comparatively high concentrations of 4.034 and 7.15 μg cm^−3^. Excluding these three data points improved the model fit (the adjusted *R*^2^ increased from 0.580 to 0.795; Akaike's information criterion dropped from 87.1 to 46.4) but did not affect model outcomes. With no obvious reason for exclusion, they were retained, and the results from the full model are reported.

### (vi) Chlorophyll *d* concentrations.

Chlorophyll *d* was detected in all coral species (Fig. 3, *Chl d*). As in the case of endolithic chlorophyll *b* concentrations, *G. retiformis* (3.393 ± 2.797 μg cm^−3^), *P. cylindrica* (5.979 ± 3.238 μg cm^−3^), and *P. mayeri* (5.622 ± 1.407 μg cm^−3^) had significantly higher concentrations than *I. palifera* (0.758 ± 0.461 μg cm^−3^) (*P* < 0.05) but were not significantly different from each other (*P* > 0.05) (Fig. 3, *Chl d*; see also Table S4). Only *P. cylindrica* was significantly greater than *M. digitata* (2.139 ± 0.980 μg cm^−3^; Fig. 3, *Chl d*; see also Table S4).

Three outliers were detected: two *G. retiformis* samples (1.038 μg cm^−3^, Cook’s distance = 0.089; 12.064 μg cm^−3^, Cook’s distance = 0.095) and one *P. mayeri* sample (0.227 μg cm^−3^, Cook’s distance = 0.312). The effect of excluding these data was considerable. In the full model, four of ten comparisons were significant (Fig. 3, *Chl d*). Excluding outliers increased the number of significant comparisons from four to seven. Given that model assumptions where still met when the outliers were included, and without a biological reason to exclude them, they were retained.

### Endolithic biomass and chlorophyll concentrations correlate with coral skeletal morphology.

**FIG 4:**
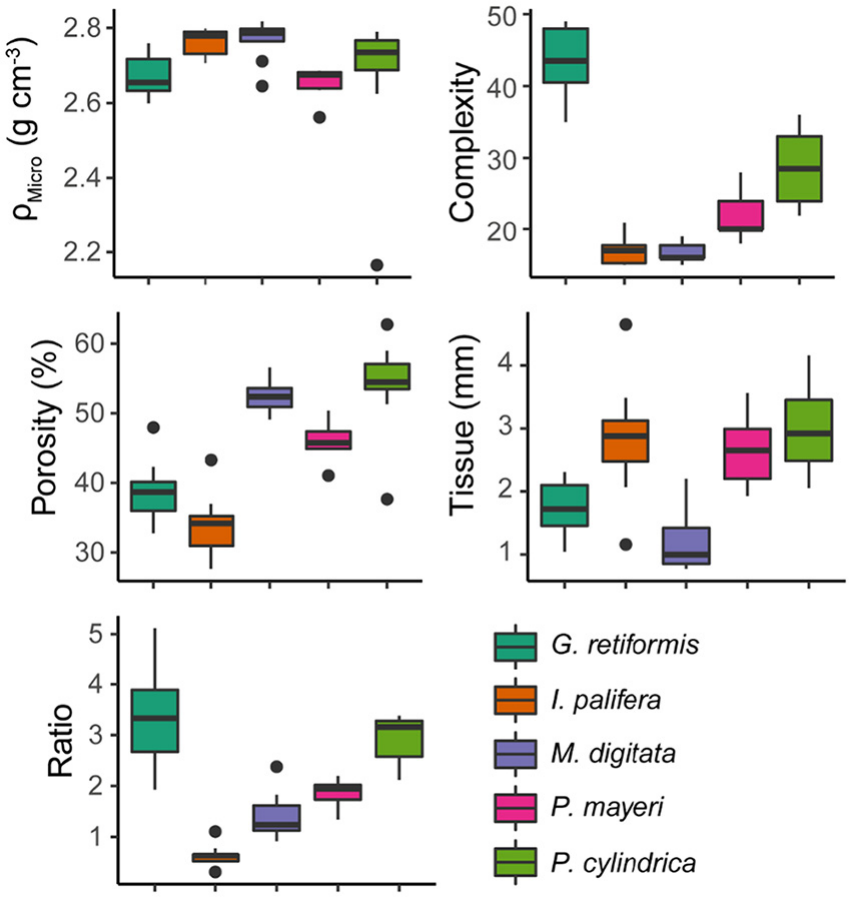
Box-and-whisker plots of the five morphological features hypothesized to influence endolithic biomass measured in each coral species: ρ_micro_ = microdensity, also known as real density, measured using Archimedean principles; complexity = corallite complexity; porosity = proportion of skeletal bulk volume that is void space; tissue = tissue thickness measured on photographed cross sections of live coral; and ratio of calice width to coenosteum width. Plots show medians and interquartile ranges (box) and the maximum/minimum (whiskers), excluding outliers (points).

**FIG 5:**
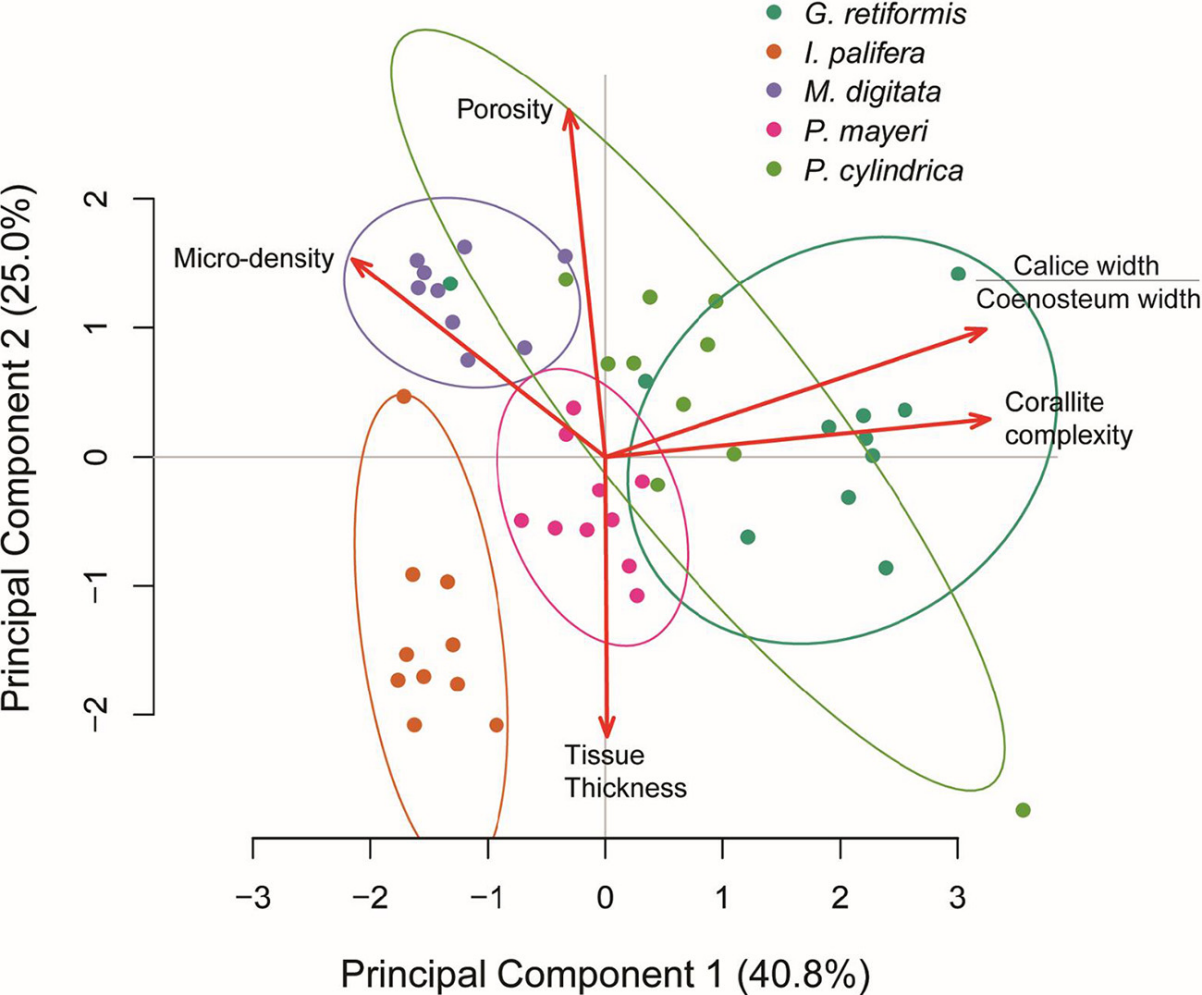
Principal component analysis biplot. Object points represent individual samples from each species, plotted by their scores for principal components 1 and 2. Red vectors represent the direction of increase for each variable that was included in the PCA. Variables were standardized by subtracting the mean and dividing by the standard deviation prior to the PCA.

The coral interspecific variation in the five morphological parameters measured in this study (Fig. 1) are shown in Fig. 4. Principal-component analysis (PCA) yielded two principal components (PC1 and PC2) that explained 40.8 and 25.0% of the variance, respectively. PC1 was loaded by corallite complexity (β = 0.643), calice/coenosteum width ratio (β = 0.636), and skeletal microdensity (β = −0.423) (Fig. 5; see also Table S5). Principal component 2 was primarily loaded by porosity (β = 0.686), tissue thickness (β = −0.554), and microdensity (β = 0.392) (Fig. 5; see also Table S5).

**FIG 6:**
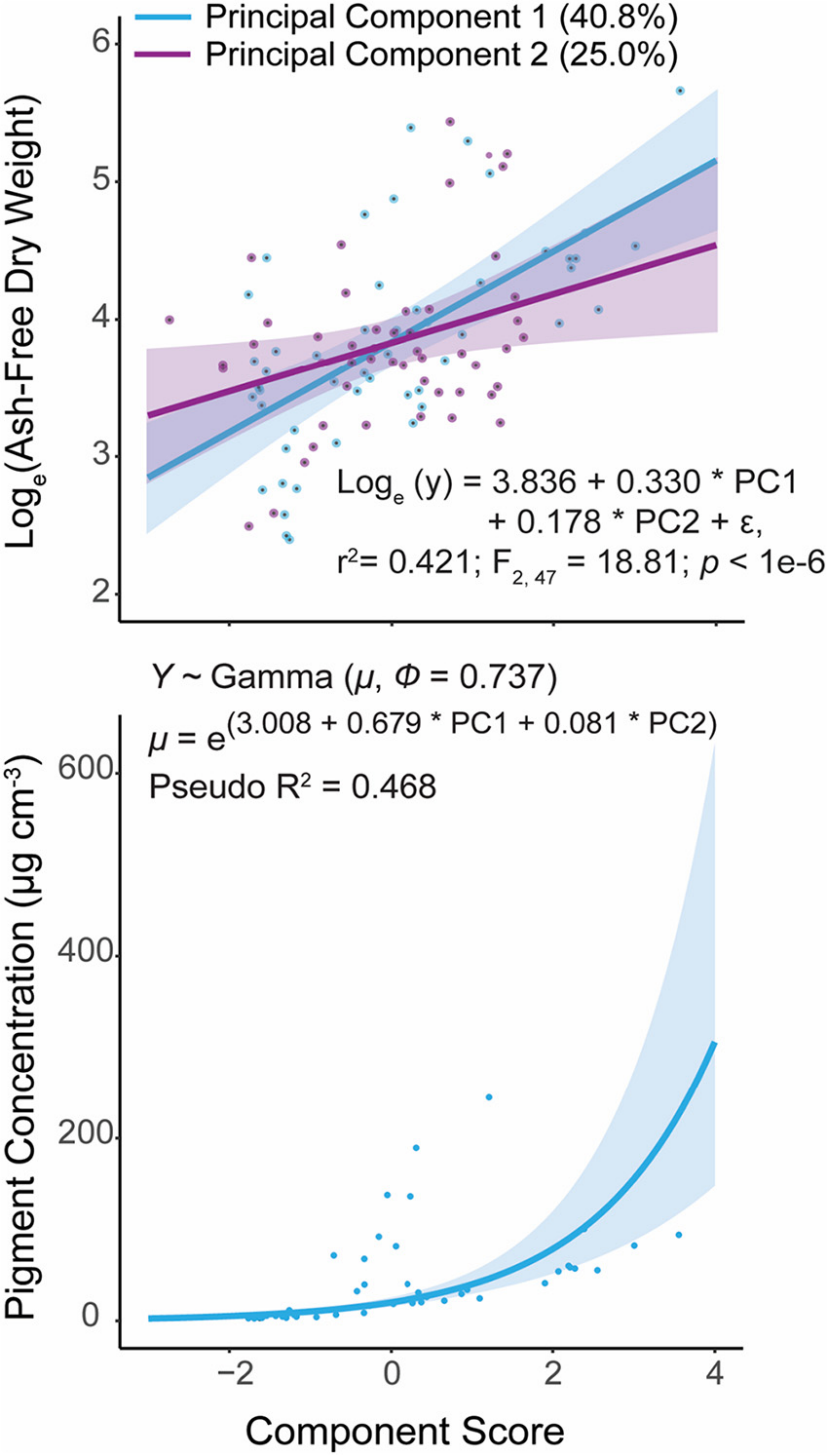
Scatterplots showing the statistically significant relationships, across all species (*n* = 50), between principal components 1 (PC1; light blue lines) and 2 (PC2; purple line) and ash-free dry weight (AFDW; mg cm^−3^) (a) and chlorophyll a concentration (μg cm^−3^) (b). These were modeled using lognormal distribution (a) and gamma distribution (b). In all cases, the regression lines represent the predicted effect of, for example, PC1 upon the value of AFDW when PC2 is held at its mean and vice versa. Insets: the linear equation used in the model as well as that model’s F-test results against a null model (a); the equation for the log-linked gamma mean (μ) and the dispersion parameter (ϕ) (b). To calculate the gamma distribution shape (α) and rate (β) parameters, α = 1/ϕ and β *=* α/μ.

When we used these principal components in a series of regression analyses, we found that PC1 was significantly correlated with endolithic microbial biomass (log-normal regression: *t* = 5.651, *P* < 0.001) and the concentrations of chlorophylls *a* (gamma regression: *t* = 7.990, *P* < 0.001), *b* (gamma regression: *t* = 7.465, *P* < 0.001), and *d* (gamma regression: *t* = 5.304, *P* < 0.001) in the endolithic microbiome (Fig. 5 and 6; see also Tables S3 and S6). PC2 was also significantly correlated with biomass (*t* = 2.383, *P* < 0.05) but only chlorophyll *d* (*t* = 3.007, *P* < 0.01). All models met their respective assumptions; the outputs of diagnostic tests are shown in Text S1 in the supplemental material.

## DISCUSSION

The endolithic biofilms of coral skeletons play a key, but as-yet-undercharacterized role in the coral metaorganism and may be important in defining how corals respond to climate change. As such, we set out to explore whether interspecific variation in coral skeletal morphology could be used to predict the biomass of and chlorophyll concentrations within the endolithic microbiome. This information could form the basis of a framework for identifying coral species, based on their morphometric traits, which are likely to be significantly impacted by endolithic community responses. Five aspects of coral morphology were measured (Fig. 1 and 4): tissue thickness, skeletal microdensity, porosity, corallite complexity, and the ratio of calice width to coenosteum width (44, 51). Several significant positive relationships were identified between biomass/chlorophyll concentrations and variables produced by a PCA of the morphological data (PC1 and PC2). These provide evidence for coral skeletal morphology structuring the biomass and phototrophic composition of endolithic biofilms.

The biomass of coral endolithic microbiomes is commonly dominated by euendolithic (i.e., “true-boring”) phototrophs such as *Ostreobium* spp.; therefore, coral skeletal microdensity was hypothesized to influence the rate of algal boring and therefore microbial biomass inside the coral skeleton. Of the five coral species we compared, we found that *G. retiformis* had the lowest skeletal microdensity (Fig. 4), the second highest endolithic biomass (Fig. 2), and the highest concentrations of chlorophylls *a* and *b* in their microbiomes (Fig. 3, *Chl a* and *Chl b*). Further, the interspecific patterns in the chlorophylls *a* and *b* (Fig. 3, *Chl a* and *Chl b*) mirror those in skeletal microdensity (Fig. 4) with less-dense skeletons harboring biofilms with higher concentrations of chlorophylls *a* and *b*. Given that only green algae (Chlorophyta) such as *Ostreobium* utilize chlorophyll *b* as an accessory pigment, this suggests that euendolithic green algae benefit from low skeletal density, possibly due to a decreased energetic cost of boring. It has also been recently proposed that *Ostreobium* spp. feed on coral skeletal organic matrices to supplement autotrophy (41). A higher relative mass of organic matrix would decrease microdensity and act as a food source for dominant microborers; therefore, this relationship may be due to more abundant food sources for *Ostreobium* in low-density skeletons. It is possible that anoxygenic green sulfur bacteria, which were found to be the dominant endolithic phototrophs in *I. palifera* from the South China Sea (25), are influencing the relationship between chlorophyll concentrations and skeletal structure for our *I. palifera* samples. However, recent work has shown that the *I. palifera* endolithic microbiome at this location is primarily composed of oxygenic phototrophs, including *Ostreobium* (52), and it is not yet known whether these two groups of phototrophs can coexist in the coral skeleton. Nonetheless, the presence of bacteriochlorophylls used by green sulfur bacteria has been found to lead to minor overestimations of chlorophyll *a* concentrations in freshwater lake sediments (53).

**FIG 7:**
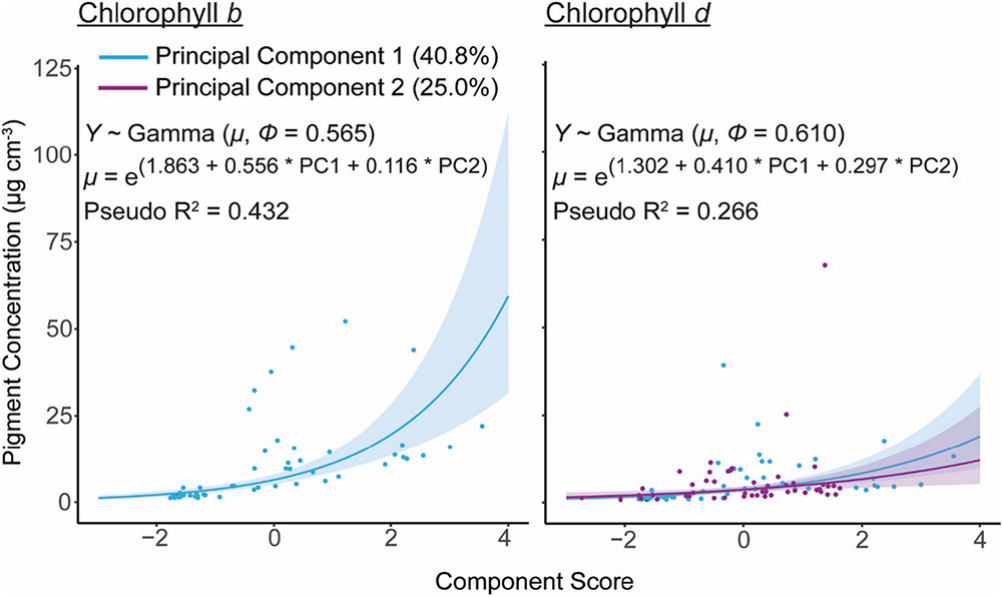
Scatterplots showing the statistically significant relationships, across all species (*n* = 50), between principal components 1 (PC1; light blue lines) and 2 (PC2; purple line), and accessory chlorophylls *b* (left) and *d* (right). These were modeled using gamma regression. In all cases, the regression lines represent the predicted effect of, for example, PC1 upon the value of AFDW when PC2 is held at its mean and vice versa. Insets show the the log-linked gamma means (μ) and dispersion parameters (ϕ) for each model. To calculate the gamma distribution shape (α) and rate (β) parameters, α = 1/ϕ and β = α/μ.

*G. retiformis* also had the largest (relative to coenosteum width) and most complex corallites, indicating that they are more effective “photon traps” than the other species studied here (44, 46, 54). In contrast, *I. palifera* and *M. digitata* have relatively high microdensities (∼2.8 g cm^−3^), low corallite complexity, and low calice/coenosteum width ratios. They also have significantly lower microbial biomasses and concentrations of chlorophylls *a* and *b* (Fig. 2 and 3, *Chl a* and *Chl b*). These observations suggest that variability in coral skeletal morphology is affecting the dominant phototrophs in the endolithic microbiome and is therefore an important driver of interspecific variability in endolithic microbial biomass. Specifically, corals with low-density skeletons that are more effective at capturing light support great abundances of green microalgae. This is reflected in the results of the principal component regression. PC1, which was loaded by the above factors (Fig. 5), is significantly positively correlated with endolithic biomass and chlorophylls *a* and *b* (Fig. 6 and 7).

*P. cylindrica* had the highest recorded endolithic biomass in our species, and yet its skeleton was more dense than its congeneric species *P. mayeri*. However, it did have a larger calice/coenosteum width ratio and more complex corallites. It also has a submassive/branching macromorphology, which makes *P. cylindrica* more effective at scattering light within the whole colony than *P. mayeri* (39). This suggests that light-capture parameters are relatively more important than microdensity in driving the observed relationship between PC1 and biomass/chlorophyll. Effective light capture at the external surface of the coral skeleton could either increase the light intensity in the endolithic environment or decrease it by scattering light more effectively in the photosymbiont-rich coral tissue. Higher light intensity inside the endolithic habitat might be expected to increase phototrophic growth. However, endolithic algae like *Ostreobium* spp. can be photochemically saturated at very low light intensities (<7 μmol photons m^−2^ s^−1^ [37]), and a recent genomic analysis revealed an absence of photoprotective genes in this shade-adapted algae (41). Therefore, they may be particularly susceptible to photodamage at high light; this could also explain why Ralph et al. (37) identified endolithic algae as boring away from the coral surface (i.e., negative phototaxis) in a shallow reef flat environment.

In contrast to microdensity and corallite morphology, porosity and tissue thickness showed no significant correlations with chlorophyll concentrations in the endolithic biofilm, and the relationship between these two variables and microbial biomass is difficult to interpret. Porosity was hypothesized as being a key driver of endolithic microbial biomass because higher porosity would represent more space for cryptoendoliths that inhabit pores and chasms in the coral skeleton. However, skeletons with high porosity also have relatively less substrate for euendoliths to colonize. Consequently, we observed pairs of species with similar porosities (e.g., *G. retiformis*/*I. palifera* or *P. cylindrica*/*M. digitata*) having very different microbial biomasses inside their skeletons (Fig. 2 and 4). Similarly, *I. palifera* and *P. cylindrica* had approximately the same tissue thicknesses but the lowest and highest biomasses, respectively (Fig. 2 and 4). Thicker tissues were hypothesized by Shashar et al. (47) as being the cause of lower light intensity measured in massive *Favia* spp. coral skeletons compared to massive *Porites* spp. skeletons. In this study, species with thin tissue (i.e., *G. retiformis* and *M. digitata*) were observed harboring biofilms with both high and low biomasses (Fig. 2 and 4).

### Conclusions.

Here, we identify correlations between interspecific variability in coral endolithic microbial biomass and skeletal morphology. Our results suggest that differences in skeletal density and a coral species’ capacity for capturing and scattering light on the outer surface of its skeleton influence the variability in skeletal microbial biomass, predominantly of dominant euendolithic green algae (e.g., *Ostreobium* spp.). Further studies exploring the responses of individual endolithic taxa to different aspects of coral skeletons are essential for defining the variable ecological role of this microbiome on coral reefs and identifying coral types that may be differentially affected (both positively and negatively) by the endolithic community.

## MATERIALS AND METHODS

### Sample collection.

In October 2019, 20 fragments of each of the following species were collected from the Heron Island reef flat (23.4423°S, 151.9148°E): *Montipora digitata*, *Porites cylindrica*, *Isopora palifera*, *Goniastrea retiformis*, and *Porites* cf. *mayeri*. Species were identified according to the descriptions by Veron and Stafford-Smith (55). All fragments were checked for the presence of macroborer burrows on the external surface before collection; if an internal borehole was seen after removing the fragment, the sample was discarded since macroborers can affect endolithic microbial biomass (56). Ten individuals per species were sampled to quantify endolithic biomass and chlorophyll concentrations and to qualitatively assess the composition of endolithic biofilms, while an additional 10 individuals per species were sampled to quantify skeletal morphological variance (tissue thickness, microstructural features, and bulk skeletal characteristics). For branching corals (*M. digitata*, *P. cylindrica*, and *I. palifera*), a branch no longer than 7 cm in length was snapped off the colony; for encrusting and massive corals (*G. retiformis* and *P. mayeri*) a piece of no more than 5 cm in diameter was removed using a hammer and chisel. For the samples used to measure skeletal morphology, the branches/fragments were sliced using a sterilized diamond-dusted circular brick saw to produce a flat cross-section of skeleton. This was washed with deionized water immediately after cutting.

### Sample processing for ash-free dry weight, chlorophyll concentration, and compound microscopy.

The 10 fragments of each species were subsequently split into three subsamples (*n *=* *150 subsamples total) for the following analyses (see Fig. S1 in the supplemental material): microbial biomass, chlorophyll concentrations, and compound microscopy. Coral tissue was removed from each subsample by airbrushing using a 12-liter aluminum compressed-air SCUBA cylinder attached to an airgun (Ozito, Australia). To maximize tissue removal from each subsample, a fragment was allowed to soak in seawater for 2 min after airbrushing. After soaking, the fragment was then airbrushed again, and this was repeated three times for each coral subsample.

10.1128/mSphere.00060-21.2 FIG S1 Schematic outlining the different analyses that were performed on each set of coral samples. Red text indicates the type of data made available at each step of the analysis. Download FIG S1, PDF file, 0.2 MB. Copyright © 2021 Fordyce et al. 2021 Fordyce et al. https://creativecommons.org/licenses/by/4.0/ This content is distributed under the terms of the Creative Commons Attribution 4.0 International license.

10.1128/mSphere.00060-21.3 FIG S2 The apparatus used for buoyant weight measurements of clean skeletons from sample set 2. This more compact design precludes the need for an under hook on the scale and therefore the scale does not need to be moved and recalibrated. It also allows the partial use of the wind shield. Measures on a four decimal place balance stabilized within 10 to 15 seconds. (c) Using skeletal samples of *Acropora aspera* (*n* = 12) and *Pocillopora damicornis* (*n* = 8), the dry mass of each sample is measured directly on the balance plate (*x* axis) and using the new apparatus (*y* axis). A perfect correlation indicates that the apparatus does not skew the accuracy of measurements. Download FIG S2, PDF file, 0.1 MB. Copyright © 2021 Fordyce et al. 2021 Fordyce et al. https://creativecommons.org/licenses/by/4.0/ This content is distributed under the terms of the Creative Commons Attribution 4.0 International license.

**(i) Measuring fragment volume using Archimedean principles.** In order to normalize measurements of biomass and chlorophyll concentrations and ensure valid interspecific comparisons, the volume of each subsample was calculated using the buoyant weight technique described by Jokiel (57), which relies upon Archimedean principles. These measurements were performed at Heron Island Research Station using a four-decimal place balance (Ohaus, NJ) positioned above a 90-liter glass aquarium filled with sand-filtered seawater from the Heron Island reef flat. The temperature of the seawater was continuously monitored and its density (ρ_SW_) calculated for every 0.1°C change in water temperature. The ρ_SW_ was calculated by comparing the weight of a reference object of known density in distilled water and seawater (51, 57). After determining the ρ_SW_, each coral sample was weighed while suspended in seawater (i.e., “wet weight”). Then, using the ρ_SW_ and the specific microdensity (ρ_Micro_) values calculated for each species from the cross-sectional samples (see “Sample processing for skeletal morphological variance” below), the equivalent dry weight is estimated:

(1) $${\text{DM}}_{\text{Coral}}=\frac{{\text{WM}}_{\text{Coral}}}{1\;-\;\left(\frac{\rho_{\text{SW}}}{\rho_{\text{Micro}}}\right)}$$

where DM represents dry mass and WM represents wet mass. Using the calculated dry weight, we then estimated the subsample skeletal biomineral volume (*V*_Bio_E_) using the following equation:

(2) $$V_{\text{Bio\_E}}=\frac{{\text{DM}}_{\text{Coral}}\;-\;{\text{WM}}_{\text{Coral}}}{\rho_{\text{Micro}}}$$

Note that these estimated biomineral volume (*V*_Bio_E_) data are distinct from the volume data used to calculate species microdensity (*V*_Bio_M_).

**(ii) Assaying microbial biomass by ash-free dry weight.** Endolithic biomass was measured in each subsample by placing it in a 50-ml centrifuge tube and dissolved by up to three acid washes using 1.6 M hydrochloric acid (HCl), similar to the method used by Fine and Loya (14). Between each wash step, the samples were centrifuged at 3,856 × *g* and 4°C for 10 min to form a microbial pellet. The supernatant with the remnant animal tissue (including skeletal organic matrix) was carefully removed using a 10-ml graduated syringe before adding more HCl. Once fully decalcified, the pellets were washed twice using 0.22-μm filtered seawater (FSW) to remove any excess HCl.

The pellet was loosened from the centrifuge tube using FSW in a transfer pipette and transferred into a sterile plastic weighing boat. It was then dried at 70°C for 18 h to remove all moisture, leaving a mixture of dried organic matter and salt crystals. This was weighed and then carefully transferred to a sterile crucible, which itself was then weighed. The process of transferring the material resulted in an average loss of 1.534 ± 0.8 mg. The crucible was then placed in a muffle furnace at 550°C for 4 h to burn off the organic matter (33). The change in mass due to burning was normalized to fragment volume to produce an estimate of endolithic biomass by ash-free dry weight (AFDW), expressed as mg cm^−3^.

**(iii) Assaying endolithic chlorophyll concentrations by spectrophotometry.** To measure the concentrations of chlorophyll pigments in the endolithic biofilm, the subsamples were first decalcified using the same procedure as described above. After decanting the FSW from the final wash, we added 10 ml of 90% acetone to extract chlorophyll from the pellet (58) and vortexed the sample for 30 s to homogenize the mixture. Cells were then placed in chilled water and fragmented using an ultrasonic cleaner (Unisonics, NSW, Australia) for 20 min in the dark. They were then vortexed for a further 30 s and chilled at 4°C for 24 h in the dark to extract the chlorophyll. After the extraction, the samples were centrifuged at 3,856 × *g* for 5 min, and the clear supernatant was used to assess chlorophyll concentration using polystyrene cuvettes and a SpectroStar Nano spectrophotometer (BMG Labtech, Ortenberg, Germany). Each subsample was measured in triplicate, and the spectrophotometer zeroed using pure 90% acetone between each subsample’s triplet of measurements.

The concentrations of chlorophylls *a*, *b*, *c*, and *d*, using absorption (optical density [OD]) values at 630 nm, 674 nm, 664 nm and 691 nm, were calculated by using the quadrichroic spectrophotometry equations from Ritchie (58). Chlorophyll concentrations were normalized to the subsample volume and are expressed as μg cm^−3^. We recognize that the HCl acidifies chlorophyll *a* to phaeophytin *a*, which lowers absorption at 664 nm and can lead to the underestimation of the chlorophyll *a* concentration by spectrophotometry (58).

**(iv) Biofilm composition by qualitative compound microscopy.** We followed adapted methods from Golubic et al. (59) to qualitatively describe the composition of endolithic biofilms in each coral species. Fragments were fixed in 4% paraformaldehyde in Ultra-Pure DNase-free 0.03 M phosphate-buffered saline (PBS) for 16 h and subsequently washed with 0.03 M PBS three times prior to decalcification with HCl to minimize the formation of formic acid. They were then decalcified as described above, using repeated washes of 1.6 M HCl. The fixed endolithic pellet was isolated through sequential washing and centrifugation. After processing, 15 ml of Ultra Pure DNase-free 0.03 M PBS was added for sample preservation. These subsamples were transported to the University of Newcastle. When imaging the biofilm, the pellet was resuspended by a combination of shaking and vortexing. A micropipette with a 1,000-μl tip attached was used to mount and smear a droplet of the mixture onto a glass microscope slide. A light microscope linked to a computer monitor (Leica, Germany) was used to image the microbes at ×100, ×200, and ×400 magnifications. Microbes were categorized into the following groups: unicellular filamentous algae, segmented filamentous algae, coccoid algae, fungi, cyanobacteria, and “other.” Where possible, *Ostreobium* spp. were identified using available resources (1, 16, 34, 60).

### Sample processing for skeletal morphological variance.

**(i) Digital photography for tissue thickness.** Photographs were taken of the cleaned cross sections, with a 15-cm ruler for scale, using a Sony RX-100 digital camera (Fig. 1). The cross-sectional photographs were first adjusted for contrast and white balanced in Adobe Lightroom. We then used ImageJ (61) to measure the thickness of the tissue layer at 10 points along the cross-section in millimeters (Fig. 1). The scale was reset for each photograph using the 15-cm ruler in the photograph. After photographing the cross-sections, these slices were soaked in 10% commercial bleach for 48 h to remove tissue and organic matter and then dried at 50°C for 48 h (62). These were then packed into hard plastic tubs with lint-free tissue and transported to the University of Newcastle.

**(ii) Measuring microdensity and porosity using Archimedean principles.** In order to measure the ρ_Micro_ and the porosity of each coral species, we followed a modified version of the method outlined by Bucher et al. (51). All mass measurements were made using a four-decimal-place Shimadzu balance (Shimadzu, Kyoto, Japan). For these measurements, we used a custom apparatus that was validated by comparing dry weight measured with or without this apparatus (see Fig. S2).

Skeletal cross sections were weighed in air (i.e., dry weight [DM_Coral_]) and then placed in a 1.5-liter vacuum chamber (Bacoeng Engineering) filled with artificial seawater with a salinity of 35‰. The chamber was evacuated of air using a single stage vacuum pump to a vacuum of −29 inHg. This draws air out of the dry skeleton and ensures no small bubbles are trapped that would otherwise skew measurements (51). The skeletons were left under vacuum for 20 h. Once saturated with seawater, they were weighed when immersed in water (i.e., wet weight [WM_Coral_]) using the apparatus in Fig. S2. Seawater temperature was monitored continuously, and the ρ_SW_ was calculated using a web-based density calculator (https://www.mt-oceanography.info/Utilities/density.html); the ρ_SW_ was recalculated for every 0.1°C change in temperature. The sample biomineral volume was then measured (*V*_Bio_M_) as follows:

(3) $$V_{\text{Bio\_M}}=\frac{{\text{DM}}_{\text{Coral}}\;-\;{\text{WM}}_{\text{Coral}}}{\rho_{\text{SW}}}$$

After calculating the *V*_Bio_M_ as shown above, this value is then used in combination with the sample dry weight to calculate the ρ_Micro_:

(4) $$\rho_{\text{Micro}}=\frac{{\text{DM}}_{\text{Coral}}}{V_{\text{Bio}}}$$

These microdensity measurements were then input into equations 1 and 2 to complete the *V*_Bio_E_ measurements used to normalize biomass and chlorophyll measurements.

After calculating the microdensity, the skeletal cross sections were washed in distilled water and dried at 50°C for 48 h before being used to measure porosity. according to the method of Bucher et al. (51), paraffin wax was first heated to 105 to 110°C. Forceps were warmed in the wax before being used to dip the skeletal samples in the wax for 1 s. After dipping, the samples were gently shaken to remove excess wax and ensure a complete wax covering of the skeleton. The wax-coated skeleton is then weighed in air and when immersed in seawater as described above. The resulting volumetric data corresponds to the bulk volume (i.e., *V*_Bulk_ = *V*_Bio_ + porosity). The porosity is then calculated as follows:

(5) $$P=V_{\text{Bulk}}\;-\;V_{\text{Bio}}$$

**(iii) Measuring corallite complexity and the ratio of calice width to coenosteum width.** According to modified methods from Swain et al. (44), we measured the corallite complexity and the calice/coenosteum width ratio as proxies for light enhancement/scattering by the coral skeleton. Corallite complexity was measured as the weighted sum of the number of septa within a corallite. The number of septa within each septal cycle was counted (Fig. 1). For *G. retiformis*, this was possible from a macrophotograph; for the other four species, septa were counted using a stereomicroscope. Septa in the primary cycle were weighted by two, while secondary septa were unweighted. The corallite complexity is the sum of all septa after weighting (*n *=* *3 corallites were measured per skeletal cross-section).

To measure the width of calices and intercorallite spaces (i.e., the coenosteum), we used a Sony RX-100 to take a macrophotograph of the external surface of each skeletal cross-section. To serve as a scale, a 15-cm ruler was positioned to be in the photograph frame and on the same plane as the surface of the samples. Using ImageJ, we measured the width of both calices/coenosteum in these images; the scale of the image was reset for each sample. For calice width, we measured the maximum width and the width of an axis at 90° to the maximum (Fig. 1); these were averaged to give a single value of calice width per corallite (*n *=* *3 corallites were measured per sample). Two measurements of coenosteum width were taken either side of each corallite for which calice width was measured (Fig. 1). The ratio of these widths was calculated; higher values equal larger calices relative to coenosteum width.

### Statistics.

All statistics were conducted using R version 3.6.0 (63). All packages used in this analysis are shown in Table S1.

10.1128/mSphere.00060-21.4 TABLE S1 R Packages used in this study. Download Table S1, DOCX file, 0.02 MB. Copyright © 2021 Fordyce et al. 2021 Fordyce et al. https://creativecommons.org/licenses/by/4.0/ This content is distributed under the terms of the Creative Commons Attribution 4.0 International license.

10.1128/mSphere.00060-21.5 TABLE S2 Presence/absence table of different microbial taxa identified, by compound microscopy (magnification: ×100 to ×400) from each coral species’ endolithic biofilms (*n* = 5 per species). Download Table S2, DOCX file, 0.02 MB. Copyright © 2021 Fordyce et al. 2021 Fordyce et al. https://creativecommons.org/licenses/by/4.0/ This content is distributed under the terms of the Creative Commons Attribution 4.0 International license.

10.1128/mSphere.00060-21.6 TABLE S3 Output for all statistical tests performed on endolithic ash-free dry weight (linear contrasts, one-way ANOVA and principal component regression). Download Table S3, XLSX file, 0.01 MB. Copyright © 2021 Fordyce et al. 2021 Fordyce et al. https://creativecommons.org/licenses/by/4.0/ This content is distributed under the terms of the Creative Commons Attribution 4.0 International license.

10.1128/mSphere.00060-21.7 TABLE S4 Output for linear contrasts applied to all one-way ANOVA models used to compare interspecific chlorophyll concentrations. Download Table S4, XLSX file, 0.01 MB. Copyright © 2021 Fordyce et al. 2021 Fordyce et al. https://creativecommons.org/licenses/by/4.0/ This content is distributed under the terms of the Creative Commons Attribution 4.0 International license.

10.1128/mSphere.00060-21.8 TABLE S5 Results of the PCA used to reduce the dimensionality of our coral morphology data for subsequent generalized linear regression; principal components 1 and 2 were used. Variables were standardized by mean centering and scaling in the process. Variable codes: CtC = calice/coenosteum width ratio; CC = corallite complexity; MD = microdensity; TT = tissue thickness; Po = porosity. Download Table S5, DOCX file, 0.01 MB. Copyright © 2021 Fordyce et al. 2021 Fordyce et al. https://creativecommons.org/licenses/by/4.0/ This content is distributed under the terms of the Creative Commons Attribution 4.0 International license.

**(i) Interspecific comparisons by one-way ANOVA.** A series of one-way ANOVAs with Tukey *post hoc* comparisons were used to test the significance of the interspecific differences observed in our measurements of AFDW and the concentrations of chlorophylls *a*, *b*, *c*, and *d*. We used Q-Q plots to check the distribution of residuals for deviations from normality and Breusch-Pagan tests (64) to test the assumption of homogeneity of variance across species. In the cases of AFDW and all chlorophyll concentrations, we were required to log transform our response variables to meet these assumptions. Where outliers were detected, using a Cook’s distance threshold of 0.08 (4/*n*) (65), we ran a model excluding these data for comparison with the original model and examined the estimates and standard errors for change. We present the data as medians ± the median absolute deviation.

**(ii) Principal component regression to correlate morphology with endolithic biomass and chlorophyll concentrations.** PCA (66) was used to extract components from the multivariate morphological data, given the high probability of these data being colinear. These components were then used in a regression analysis to identify possible correlations between morphological data and endolithic biomass/chlorophyll concentrations. Morphological variables were first centered and scaled to account for differences in each variable’s scale by subtracting the mean and dividing by the standard deviation. Principal component scores and factor loadings were calculated using the *princomp* function (63), while eigenvalues were extracted using the factoextra package (67). We visualized the data using the pca3d package (68).

The first two principal components (PC1 and PC2) were selected as independent variables in generalized linear models where the response variables were AFDW or concentrations of chlorophylls *a*, *b*, and *d*. For AFDW, a natural logarithm transformation was applied to the response variables to meet the assumptions that residuals are normally distributed, and variance is homogeneous across the scale of the predictor. Data points with Cook’s distance greater than 4/*n* were considered outliers (65) and investigated further. Regression models were run with or without the outliers, and the results were compared.

For chlorophylls *a*, *b*, and *d*, we used the fitdistrplus package (69) to compare the goodness of fit for normal, lognormal, and gamma distributions. This highlighted a generalized linear model with gamma distribution as the best fit for all chlorophyll pigment types. We used the lme4 package (70) to run these and diagnosed model fit using the DHARMa package (71) to test for over- or underdispersion (nonparametric dispersion test, fitted versus simulated residuals) and check that the deviance residuals were normally distributed on the log scale (Q-Q plot and KS test). Zero centering of the deviance residuals was verified graphically. We also calculated the pseudo-*R*^2^ value as follows:

(6) $$\text{Pseudo−}R^{2}\;=\;1\;-\;\left(\frac{\text{Model deviance}}{\text{Null deviance}}\right)$$

Outliers were detected using the above procedure for detection and testing.

### Data availability.

All data and associated code are available on Github at https://github.com/GusFordyce/Coral-morphometrics-and-endolithic-biomass.

10.1128/mSphere.00060-21.9 TABLE S6 Output for principal component regression models applied chlorophyll concentrations. Download Table S6, XLSX file, 0.01 MB. Copyright © 2021 Fordyce et al. 2021 Fordyce et al. https://creativecommons.org/licenses/by/4.0/ This content is distributed under the terms of the Creative Commons Attribution 4.0 International license.
