# Supplementary material for: Light Capture, Skeletal Morphology, and the Biomass of Corals’ Boring Endoliths
Source: mSphere. 2021 Feb 24;6(1):e00060-21. doi: 10.1128/mSphere.00060-21 (PMC8544882; doi:10.1128/mSphere.00060-21)
Supplement: TEXT S1 [file msphere.00060-21-s0001.pdf]

Supplementary Text 1

Validation of customised buoyant weight set-up.

Methods

We used a combination of correlation analyses and paired Student's t-tests to identify possible weighing biases introduced by the buoyant weight apparatus (Fig. S2) (Spearman's ranked correlation, n= 20).

Results

A perfect correlation (Spearman's ρ = 1; Fig. S2) between coral dry weights measured by our apparatus and those measured directly on the weighing plate indicated no bias was introduced by our apparatus.

Output of diagnostic tests applied to gamma regression models used for the principal component regression of chlorophyll a, b and d concentrations.

Chlorophyll *a*

The deviance residuals from the gamma regression were zero-centred and normally distributed on the log scale (KS test = 0.120, p = 0.431), nor was there significant over- or under-dispersion (Dispersion test = 0.939, p = 0.892). One *P. cylindrica* sample was detected as an outlier (Cook's distance = 0.169) but excluding this did not affect model outcomes nor the standard errors of the model estimates.

Chlorophyll *b*

The deviance residuals from the chlorophyll b gamma regression model were normally distributed (KS test = 0.103, p=0.625) and zero-centred, and the model was neither over- nor under-dispersed (Dispersion test = 1.025, p = 0.716). Two *P.*

cylindrica samples (8.611 and 17.792 µg cm^{-3}) had Cook's distances greater than 0.08 (0.159 and 0.109 respectively) but their exclusion did not affect overall model outcomes nor the standard errors of model estimates.

Chlorophyll *d*

The gamma regression model using PC1 and PC2 as independent variables met the assumptions of zero-centring and normality in the distribution of deviance residuals (KS test = 0.087, p = 0.822) as well as a lack of significant over- or under-dispersion (Dispersion test = 1.030, p = 0.696). Two outliers were present, one *P. cylindrica* sample (16.922 µg cm^{-3}, Cook's distance = 0.196) and one *G. retiformis* sample (1.974 µg cm^{-3}, Cook's distance = 0.084). These affected neither the model outcomes for the standard errors of the coefficients.
