# Supplementary material for: Light Capture, Skeletal Morphology, and the Biomass of Corals’ Boring Endoliths
Source: mSphere. 2021 Feb 24;6(1):e00060-21. doi: 10.1128/mSphere.00060-21 (PMC8544882; doi:10.1128/mSphere.00060-21)
Supplement: FIG S1 [file msphere.00060-21-sf001.pdf]

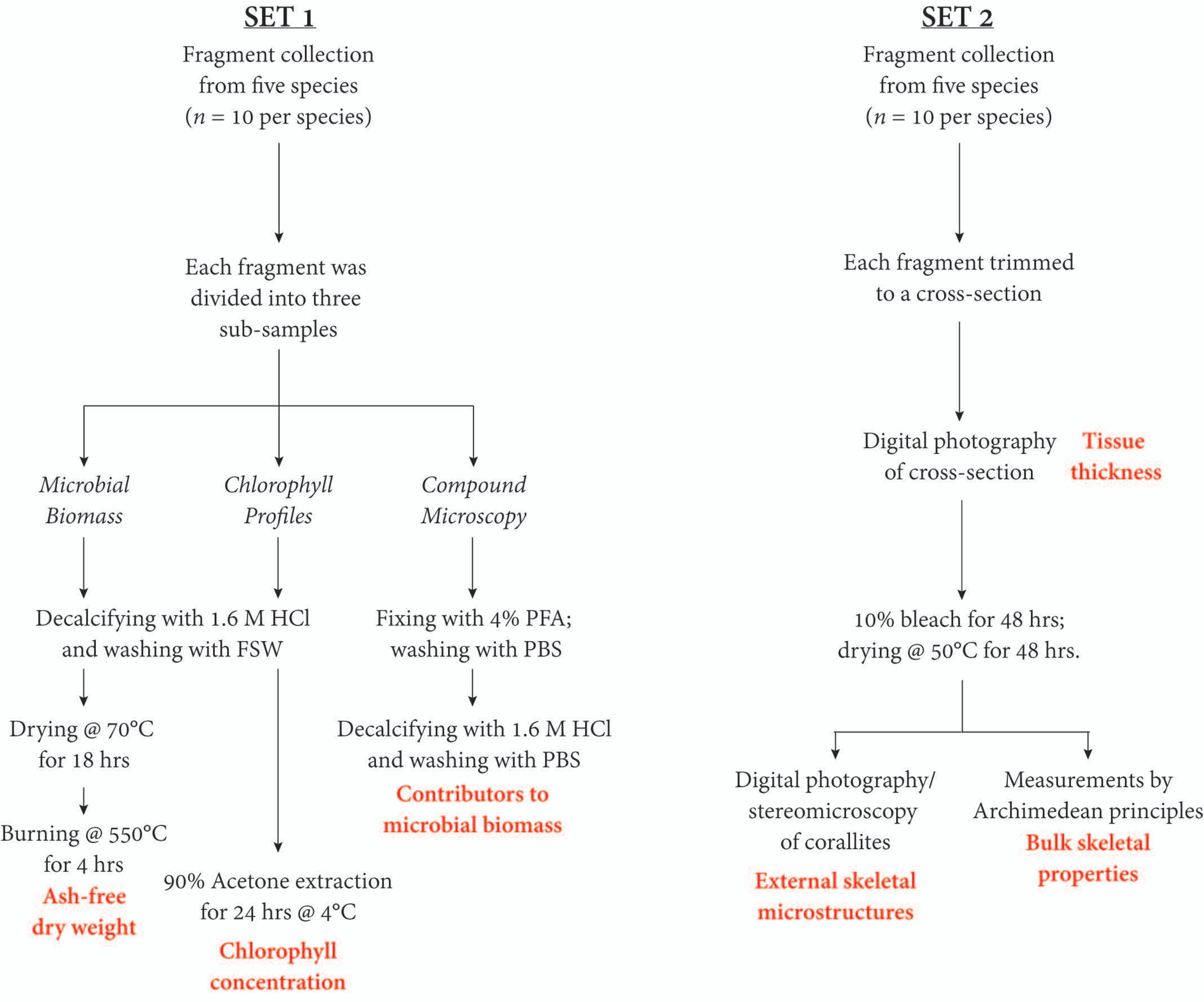
SET 1
Fragment collection from five species (n = 10 per species)
Each fragment was divided into three sub-samples
Microbial Biomass
Chlorophyll Profiles
Compound Microscopy
Decalcifying with 1.6 M HCl and washing with FSW
Fixing with 4% PFA; washing with PBS
Drying @ 70°C for 18 hrs
Decalcifying with 1.6 M HCl and washing with PBS
Contributors to microbial biomass
Burning @ 550°C for 4 hrs
Ash-free dry weight
90% Acetone extraction for 24 hrs @ 4°C
Chlorophyll concentration
SET 2
Fragment collection from five species (n = 10 per species)
Each fragment trimmed to a cross-section
Digital photography of cross-section
Tissue thickness
10% bleach for 48 hrs; drying @ 50°C for 48 hrs.
Digital photography/ stereomicroscopy of corallites
External skeletal microstructures
Measurements by Archimedean principles
Bulk skeletal properties
