# Supplementary figures and images for: Light Capture, Skeletal Morphology, and the Biomass of Corals’ Boring Endoliths
Source: mSphere. 2021 Feb 24;6(1):e00060-21. doi: 10.1128/mSphere.00060-21 (PMC8544882; doi:10.1128/mSphere.00060-21)

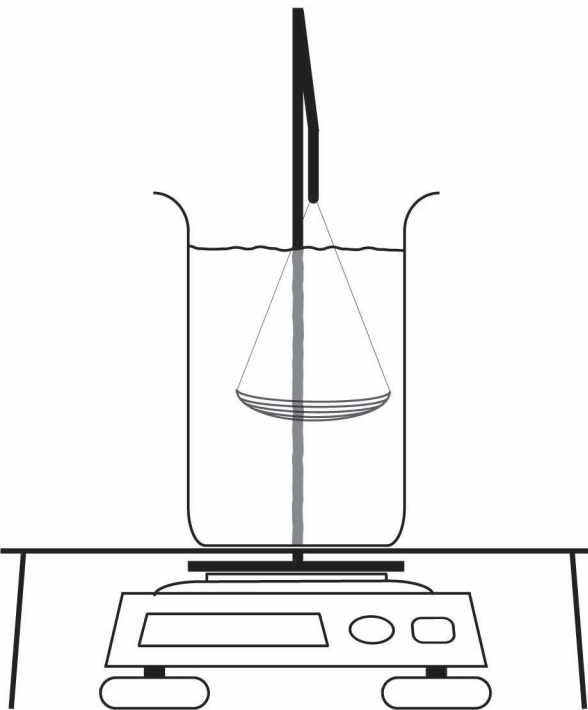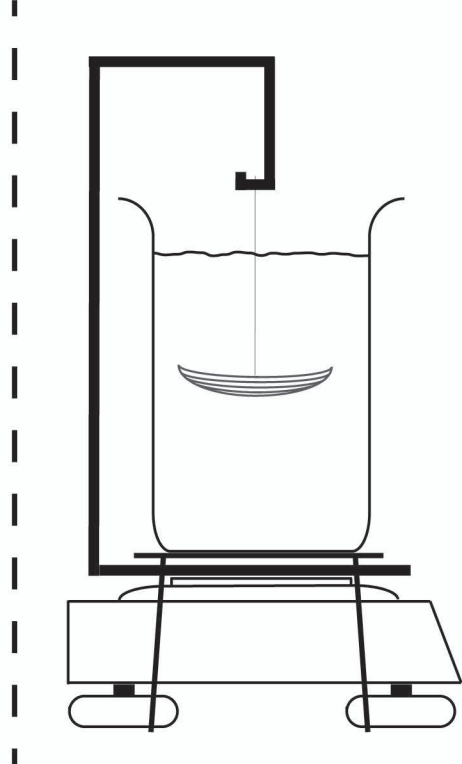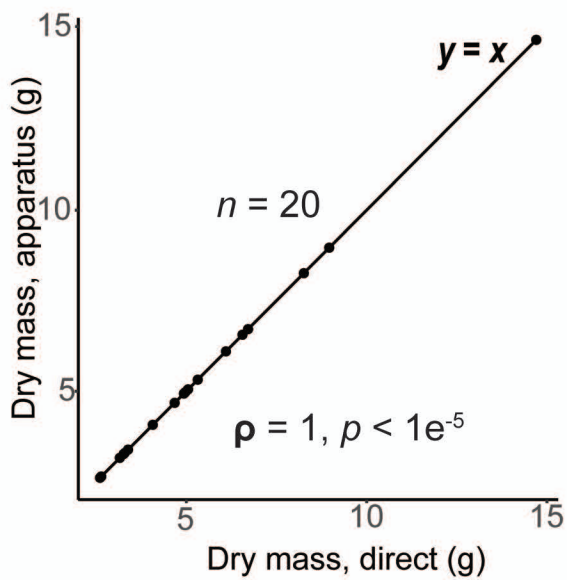

Supplement: FIG S2 [file msphere.00060-21-sf002.pdf]
