# Supplementary material for: Light Capture, Skeletal Morphology, and the Biomass of Corals’ Boring Endoliths
Source: mSphere. 2021 Feb 24;6(1):e00060-21. doi: 10.1128/mSphere.00060-21 (PMC8544882; doi:10.1128/mSphere.00060-21)
Supplement: TABLE S1 [file msphere.00060-21-st001.docx]

|  | | |
| --- | --- | --- |
| **Package** | **Author(s)** | **Justification** |
| DHARMa | (Hartig, 2017) | Used in the model diagnostics for gamma regression models |
| dplyr | Wickham, Francois, Henry, and Müller (2015) | Data manipulation |
| emmeans | Lenth (2018) | Planned Tukey Contrasts, Estimated Marginal Means |
| factoextra | (Kassambara, 2017) | Creating biplots for principal component analysis |
| fitdistrplus | Delignette-Muller and Dutang (2015) | Model distribution fitting |
| gdata | Warnes et al. (2014) | Data manipulation |
| ggfortify | (Tang, Horikoshi, & Li, 2016) | Ggplot2 add-on; Data exploration and plotting of multivariate models |
| ggplot2 | Wickham (2016) | Data plotting and visualisation |
| gridExtra | Auguie (2016) | ggplot2 add-on for multi-facet plots |
| Hmisc | Harrell Jr and Dupont (2008) | Correlation matrices |
| jtools | (Long, 2018) | Exploration and plotting of generalised linear model predictions. |
| Lattice | Sarkar (2008) | Graphical data exploration |
| lme4 | Bates, Mächler, Bolker, and Walker (2014) | Linear Mixed Effects Models |
| lmerTest | (Kuznetsova, Brockhoff, & Christensen, 2017) | Add on to lme4 to aid in diagnostic testing |
| lmtest | Zeileis and Hothorn (2002) | Regression diagnostics |
| MASS | Venables and Ripley (2013) | Generalised linear models |
| Pca3d | (Weiner, 2015) | 3D visualisation of PCA results (see Supp Fig.) |
| plyr | Wickham (2011) | Data manipulation |
| reshape2 | Wickham (2007) | Data manipulation |
| RColorBrewer | (Neuwirth & Neuwirth, 2014) | Colour-blind friendly colour palettes for ggplot2 |
| rgl | (Adler, Nenadic, & Zucchini, 2003) | Exporting 3D PCA plots as interactive WebGL files (see Supp. Fig.) |
| usdm | Naimi (2015) | Variance inflation factor for regression |

Adler, D., Nenadic, O., & Zucchini, W. (2003). *Rgl: A r-library for 3d visualization with opengl.* Paper presented at the Proceedings of the 35th Symposium of the Interface: Computing Science and Statistics, Salt Lake City.

Auguie, B. (2016). gridExtra: miscellaneous functions for “grid” graphics. *R package version, 2*(1), 242.

Bates, D., Mächler, M., Bolker, B., & Walker, S. (2014). Fitting linear mixed-effects models using lme4. *arXiv preprint arXiv:1406.5823*.

Delignette-Muller, M. L., & Dutang, C. (2015). fitdistrplus: An R package for fitting distributions. *Journal of Statistical Software, 64*(4), 1-34.

Harrell Jr, F. E., & Dupont, C. (2008). Hmisc: harrell miscellaneous. *R package version, 3*(2).

Hartig, F. (2017). DHARMa: residual diagnostics for hierarchical (multi-level/mixed) regression models. *R package version 0.1, 5*.

Kassambara, A. (2017). *Practical guide to principal component methods in R: PCA, M (CA), FAMD, MFA, HCPC, factoextra* (Vol. 2): STHDA.

Kuznetsova, A., Brockhoff, P. B., & Christensen, R. H. B. (2017). lmerTest package: tests in linear mixed effects models. *Journal of statistical software, 82*(13).

Lenth, R. (2018). Estimated Marginal Means, aka Least Squares Means. R Package version 1.1. In.

Long, J. (2018). jtools: analysis and presentation of social scientific data. R package version 1.1. 1. In.

Naimi, B. (2015). usdm: Uncertainty analysis for species distribution models. *R package version, 1*, 1-12.

Neuwirth, E., & Neuwirth, M. E. (2014). Package ‘RColorBrewer’. *URL:* [*https://cran*](https://cran)*. r-project. org/web/packages/RColorBrewer/index. html*.

Sarkar, D. (2008). *Lattice: multivariate data visualization with R*: Springer Science & Business Media.

Tang, Y., Horikoshi, M., & Li, W. (2016). ggfortify: unified interface to visualize statistical results of popular R packages. *The R Journal, 8*(2), 478-489.

Venables, W. N., & Ripley, B. D. (2013). *Modern applied statistics with S-PLUS*: Springer Science & Business Media.

Warnes, G. R., Bolker, B., Gorjanc, G., Grothendieck, G., Korosec, A., Lumley, T., . . . Rogers, J. (2014). gdata: Various R programming tools for data manipulation. *R package version, 2*(3), 35.

Weiner, J. (2015). pca3d: Three dimensional PCA plots. *R package version 0.8, 484*.

Wickham, H. (2007). Reshaping data with the reshape package. *Journal of Statistical Software, 21*(12), 1-20.

Wickham, H. (2011). The split-apply-combine strategy for data analysis. *Journal of Statistical Software, 40*(1), 1-29.

Wickham, H. (2016). *ggplot2: elegant graphics for data analysis*: Springer.

Wickham, H., Francois, R., Henry, L., & Müller, K. (2015). dplyr: A grammar of data manipulation. *R package version 0.4, 3*.

Zeileis, A., & Hothorn, T. (2002). Diagnostic checking in regression relationships.
