# Supplementary material for: Light Capture, Skeletal Morphology, and the Biomass of Corals’ Boring Endoliths
Source: mSphere. 2021 Feb 24;6(1):e00060-21. doi: 10.1128/mSphere.00060-21 (PMC8544882; doi:10.1128/mSphere.00060-21)
Supplement: TABLE S2 [file msphere.00060-21-st002.docx]

| Coral Species \| Sample No. | *Ostreobium* spp. | Filamentous, unicellular algae (general) | Filamentous, segmented algae | Coccoid green algae | Cyanobacteria | Fungi | Other | |
| --- | --- | --- | --- | --- | --- | --- | --- | --- |
| *G. retiformis* \| 1 | ✓ | ✓ | ✓ | ✓ | ✓ | ✓ | ✓ | Protists |
| *G. retiformis* \| 2 | ✓ | ✓ | ✓ |  |  | ✓ |  |  |
| *G. retiformis* \| 3 | ✓ | ✓ | ✓ |  |  |  |  |  |
| *G. retiformis* \| 4 | ✓ | ✓ | ✓ | ✓ | ✓ | ✓ |  |  |
| *G. retiformis* \| 5 | ✓ | ✓ | ✓ | ✓ | ✓ |  |  |  |
| *P. mayeri* \| 1 | ✓ | ✓ |  |  | ✓ | ✓ |  |  |
| *P. mayeri* \| 2 | ✓ | ✓ |  |  | ✓ |  |  |  |
| *P. mayeri* \| 3 | ✓ | ✓ | ✓ | ✓ | ✓ | ✓ | ✓ | Nematode |
| *P. mayeri* \| 4 | ✓ | ✓ |  | ✓ | ✓ |  |  |  |
| *P. mayeri* \| 5 | ✓ | ✓ | ✓ | ✓ |  | ✓ | ✓ | Nematode |
| *P. cylindrica* \| 1 | ✓ | ✓ |  |  |  |  | ✓ | Protists |
| *P. cylindrica* \| 2 | ✓ | ✓ |  |  | ✓ | ✓ | ✓ | Nematode; Protists |
| *P. cylindrica* \| 3 | ✓ | ✓ |  |  | ✓ | ✓ | ✓ | Nematode; Protists; Red algae |
| *P. cylindrica* \| 4 | ✓ | ✓ | ✓ |  | ✓ | ✓ | ✓ | Nematode; Protists |
| *P. cylindrica* \| 5 | ✓ | ✓ |  |  | ✓ | ✓ | ✓ | Protists |
| *I. palifera* \| 1 | ✓ | ✓ |  | ✓ |  |  | ✓ | Protists |
| *I. palifera* \| 2 | ✓ | ✓ |  |  |  |  |  |  |
| *I. palifera* \| 3 | ✓ | ✓ |  |  | ✓ |  |  |  |
| *I. palifera* \| 4 | ✓ | ✓ |  |  | ✓ |  |  |  |
| *I. palifera* \| 5 | ✓ | ✓ |  |  | ✓ |  |  |  |
| *M. digitata* \| 1 | ✓ | ✓ |  |  | ✓ |  |  |  |
| *M. digitata* \| 2 | ✓ | ✓ |  |  |  |  |  |  |
| *M. digitata* \| 3 | ✓ | ✓ | ✓ |  |  |  |  |  |
| *M. digitata* \| 4 | ✓ | ✓ |  |  | ✓ |  |  |  |
| *M. digitata* \| 5 | ✓ | ✓ |  |  | ✓ |  |  |  |
