# Supplementary material for: Light Capture, Skeletal Morphology, and the Biomass of Corals’ Boring Endoliths
Source: mSphere. 2021 Feb 24;6(1):e00060-21. doi: 10.1128/mSphere.00060-21 (PMC8544882; doi:10.1128/mSphere.00060-21)
Supplement: TABLE S5 [file msphere.00060-21-st005.docx]

| Eigenvector | Eigenvalue | Variance Explained (%) | Loadings | |
| --- | --- | --- | --- | --- |
|  |  |  | *Variable (s)* | *Standardised co-efficients* |
| Principal Component 1 | 2.042 | 40.838 | CtC  CC  MD | 0.636  0.643  -0.423 |
| Principal Component 2 | 1.251 | 25.018 | CtC  TT  Po  MD | 0.252  -0.554  0.686  0.392 |
| Principal Component 3 | 0.928 | 18.559 | CtC  TT  CC  Po | 0.165  0.771  -0.135  0.598 |
| Principal Component 4 | 0.668 | 13.367 | CtC  TT  CC  Po  MD | 0.150  0.309  0.355  -0.309  0.813 |
| Principal Component 5 | 0.111 | 2.218 | CtC  CC  Po | 0.694  -0.661  -0.269 |
